# Models for Count Data With an Application to Healthy Days Measures: Are You Driving in Screws With a Hammer?

**DOI:** 10.5888/pcd11.130252

**Published:** 2014-03-27

**Authors:** Hong Zhou, Paul Z. Siegel, John Barile, Rashid S. Njai, William W. Thompson, Charlotte Kent, Youlian Liao

**Affiliations:** Author Affiliations: Paul Z. Siegel, Rashid S. Njai, Charlotte Kent, Youlian Liao, William W. Thompson, Centers for Disease Control and Prevention, Atlanta, Georgia; John Barile, University of Hawaii at Manoa, Manoa, Hawaii.

## Abstract

**Introduction:**

Count data are often collected in chronic disease research, and sometimes these data have a skewed distribution. The number of unhealthy days reported in the Behavioral Risk Factor Surveillance System (BRFSS) is an example of such data: most respondents report zero days. Studies have either categorized the Healthy Days measure or used linear regression models. We used alternative regression models for these count data and examined the effect on statistical inference.

**Methods:**

Using responses from participants aged 35 years or older from 12 states that included a homeownership question in their 2009 BRFSS, we compared 5 multivariate regression models — logistic, linear, Poisson, negative binomial, and zero-inflated negative binomial — with respect to 1) how well the modeled data fit the observed data and 2) how model selections affect inferences.

**Results:**

Most respondents (66.8%) reported zero mentally unhealthy days. The distribution was highly skewed (variance = 58.7, mean = 3.3 d). Zero-inflated negative binomial regression provided the best-fitting model, followed by negative binomial regression. A significant independent association between homeownership and number of mentally unhealthy days was not found in the logistic, linear, or Poisson regression model but was found in the negative binomial model. The zero-inflated negative binomial model showed that homeowners were 24% more likely than nonowners to have excess zero mentally unhealthy days (adjusted odds ratio, 1.24; 95% confidence interval, 1.08–1.43), but it did not show an association between homeownership and the number of unhealthy days.

**Conclusion:**

Our comparison of regression models indicates the importance of examining data distribution and selecting models with appropriate assumptions. Otherwise, statistical inferences might be misleading.

## MEDSCAPE CME

Medscape, LLC is pleased to provide online continuing medical education (CME) for this journal article, allowing clinicians the opportunity to earn CME credit.

This activity has been planned and implemented in accordance with the Essential Areas and policies of the Accreditation Council for Continuing Medical Education through the joint sponsorship of Medscape, LLC and Preventing Chronic Disease. Medscape, LLC is accredited by the ACCME to provide continuing medical education for physicians.

Medscape, LLC designates this Journal-based CME activity for a maximum of 1 **AMA PRA Category 1 Credit(s)™**. Physicians should claim only the credit commensurate with the extent of their participation in the activity.

All other clinicians completing this activity will be issued a certificate of participation. To participate in this journal CME activity: (1) review the learning objectives and author disclosures; (2) study the education content; (3) take the post-test with a 70% minimum passing score and complete the evaluation at www.medscape.org/journal/pcd (4) view/print certificate.

**Release date: March 27, 2014; Expiration date: March 27, 2015**

### Learning Objectives

Upon completion of this activity, participants will be able to:

- Distinguish characteristics of different tools for data analysis
- Analyze how data regarding self-reported health can be skewed in the Behavioral Risk Factor Surveillance System (BRFSS) survey
- Evaluate results of different evaluation tools on count data from the BRFSS survey

**EDITORS**

Ellen Taratus, Editor, *Preventing Chronic Disease*. Disclosure: Ellen Taratus has disclosed no relevant financial relationships.

**CME AUTHOR**

Charles P. Vega, MD, Associate Professor and Residency Director, Department of Family Medicine, University of California, Irvine. Disclosure: Charles P. Vega, MD, has disclosed no relevant financial relationships.

**AUTHORS AND CREDENTIALS**

Disclosures: Hong Zhou, Paul Z. Siegel, Rashid S. Njai, Charlotte Kent, Youlian Liao, William W. Thompson, and John Barile have disclosed no relevant financial relationships.

Hong Zhou, MS, MPH, Division of Health Informatics and Surveillance, Center for Surveillance, Epidemiology and Laboratory Services, Centers for Disease Control and Prevention, Atlanta, Georgia. Paul Z. Siegel, MD, MPH; Rashid S. Njai, PhD; Charlotte Kent, PhD; and Youlian Liao, MD, National Center for Chronic Disease Prevention and Health Promotion, Centers for Disease Control and Prevention, Atlanta, Georgia. William W. Thompson, PhD, National Center on Birth Defects and Developmental Disabilities, Centers for Disease Control and Prevention, Atlanta, Georgia. John Barile, PhD, Department of Psychology, University of Hawaii at Manoa, Manoa, Hawaii.

## Introduction

Researchers of chronic disease often gather data that are measured on a continuum rather than as a “present–absent” or “yes–no” dichotomy. Examples include the following: episodes of a symptom; number of sick days, cigarettes smoked, or alcoholic drinks consumed; measures of health care use, such as number of doctor visits or days of hospitalization; and costs incurred (in dollars). Such measures are referred to as “count” data; that is, the observations can have only nonnegative integer values (0, 1, 2, 3, . . . ). Such data are most often gathered during a specified period of time (eg, the past month or year). For some of these measures, most study participants may have a zero count (eg, no episode of a symptom, no cigarettes smoked, no use of health care services). These data are typically not normally distributed, and the positive skew in their distribution cannot be resolved by data transformation. The Centers for Disease Control and Prevention’s (CDC’s) health-related quality of life (HRQOL) Healthy Days measure (1) is an example of such count data.

The Behavioral Risk Factor Surveillance System (BRFSS) questionnaire includes an HRQOL section composed of 3 questions related to respondents’ healthy days. These questions ask respondents to report the number of days in the previous 30 days when 1) their physical health was not good, 2) their mental health was not good, and 3) poor physical or mental health kept them from doing their usual activities (2). Responses to the Healthy Days questions are count data because the response must be an integer. For each of the Healthy Days questions, most respondents report zero days (2), and most of the nonzero responses are concentrated in the left side of the distribution, producing a skewed distribution with large variance.

Two simple and familiar methods have often been used to analyze Healthy Days data. The first categorizes the data into 2 (eg, ≥14 vs <14 d) (3–6) or more (eg, 0 d, 1–13 d, and ≥14 d) categories (7). Although categorizing these data may simplify the statistical analyses, there may be drawbacks (8–12), including the loss of information and power (8,10,11). Categorization does not make use of within-category information, and all participants above or below a particular cut point are treated equally even though the outcome among participants within a particular category may vary significantly: for example, 1 bad mental health day in the previous 30 days is quite different from 12 bad days, even though 1 and 12 are both in the category of less than 14 days. In addition, the selection of cut points is often arbitrary, making it difficult to compare results among studies and hampering meta-analysis. Furthermore, categorizing a continuous variable may bias results (9,12).

The second most common method of analyzing the association between various risk factors and the number of reported physically and mentally unhealthy days uses linear regression models and keeps the outcome in its original scale of 0 to 30 days (13–15). These approaches often violate the assumption of normal distribution of errors, which can distort true relationships and render significance tests invalid (16,17). Several regression models are appropriate for analyzing count data, including Poisson, negative binomial, zero-inflated Poisson, and zero-inflated negative binomial regression (18); however, they have not been used widely in analyzing Healthy Days data (19).

This study used data from the 12 states that included a question on homeownership in their 2009 BRFSS to examine the independent relationship between homeownership and number of mentally unhealthy days. Studies have shown that homeownership is associated with several health outcomes (20,21), but we are not aware of any study that has examined the relationship between homeownership and HRQOL. Our objective was to determine whether using different analytic methods produced different findings. We compared 5 multivariate regression models — logistic, linear, Poisson, negative binomial, and zero-inflated negative binomial — with respect to 1) how well the modeled data fit the observed data and 2) how model selections affect inferences.

## Methods

### Data source

BRFSS is a state-based system of annual health surveys (22). Data are collected monthly in all 50 states, the District of Columbia, Puerto Rico, the Virgin Islands, and Guam. More than 300,000 interviews are completed each year. The survey uses a multistage design based on random-digit–dialing methods to gather a representative sample from each state’s noninstitutionalized civilian resident population aged 18 years or older. The BRFSS questionnaire consists of core component questions asked in all states and optional questions (modules) asked at the discretion of the states. In 2009, a social context module including a homeownership question was asked in 12 states: Alabama, Arkansas, California, Hawaii, Illinois, Kansas, Louisiana, Nebraska, New Mexico, Oklahoma, South Carolina, and Wisconsin. Response rates for the 12 states included in this analysis had a median of 59% and ranged from 43% to 67%.

The independent variable for this study was homeownership, based on the following question in the BRFSS: “Do you own or rent your home?” The response options are own, rent, or other arrangement (such as group home or staying with friends or family without paying rent). We classified respondents who rented a home or lived by other arrangement as non-homeowners. The outcome measure was the number of days reported by respondents to the question: “Now thinking about your mental health, which includes stress, depression, and problems with emotions, for how many days during the past 30 days was your mental health not good?” Covariates included age, sex, race/ethnicity, education, household income, marital status, household size, and employment status. The 2009 BRFSS questionnaire is available at www.cdc.gov/brfss/questionnaires/pdf-ques/2009brfss.pdf.

### Data analysis

There were 68,258 adults aged 18 or older who responded to both the homeownership and mentally unhealthy days questions in the 12 states. We limited the analysis to the 60,113 people aged 35 or older, because those younger than 35 were unlikely to own a home. We excluded 550 (0.9%) people who had missing data for any of these covariates: education, marital status, household size, and employment status. People with missing data on household income (n = 6,582, 7.5%) were classified as a separate category (“unknown”) and were not excluded from the analysis. The analyzed sample included 59,563 adults (22,568 men and 36,995 women).

We first examined the distribution of mentally unhealthy days, including the frequency of zero, mean, median, skew, and variance. We then examined the associations between homeownership and number of mentally unhealthy days by using 5 models:

**Model 1: Logistic regression.** This model has been used in previous HRQOL studies (3,5). As was done in previous studies (3–5), we dichotomized the data into 2 categories of mentally unhealthy days (≥14 d vs <14 d).

**Model 2: Ordinary least-squares (OLS) linear regression. **This model also has been used in previous HRQOL studies (13–15). This is not a primary model for count data because standard OLS regression makes key assumptions about the data, such as the linearity of the relationship between the predictors and the outcome variable and normality of errors (residuals) (23).

**Model 3: Poisson regression.** This regression model is popular and also the simplest regression model for count data. It assumes a Poisson distribution, characterized by a positive skew and a variance that equals the mean (18).

**Model 4: Negative binomial regression.** This model is used when count data are overdispersed (ie, when the variance exceeds the mean). Overdispersion, caused by heterogeneity or an excess number of zeros (or both) to some degree is inherent to most Poisson data (18). We tested alpha (α), an overdispersion parameter in the negative binomial model and also used the likelihood ratio test to determine a preference between the Poisson regression and the negative binomial regression.

**Model 5: Zero-inflated negative binomial regression.** This model provides a way of modeling the excess number of zeros (with respect to a Poisson distribution or negative binomial distribution) in addition to allowing for count data that are skewed and overdispersed. It is a 2-component model, which combines the logistic regression model and the negative binomial model. The first component of the model, logistic regression for excess zeros, predicts the probability of having excess zero unhealthy days. The second component, negative binomial regression for the full range of counts, including random zeros, predicts the frequency of the unhealthy day count (18). We used the Vuong test, a likelihood-ratio–based test, to compare the zero-inflated negative binomial model with an ordinary negative binomial regression model (24). A significant *z*-test indicates that the zero-inflated model is preferred.

For each model, we plotted the sample (observed) percentage distribution of the number of unhealthy days (from 0 to 30) against the distribution predicted by the model. If the percentage distribution predicted by a model closely matched the observed distribution in the plot, the model was considered a good fit to the data.

In the modeling, we simultaneously adjusted for age (35–44, 45–54, 55–64, and ≥65), sex, race and ethnicity (non-Hispanic white, non-Hispanic black, Hispanic, and all others), education level (less than high school, high school graduate to <4 y of college, and ≥4 y of college ), household income (<25,000, 25,000 to <50,000, ≥50,000, and unknown), marital status (married, divorced/widowed/separated, and never married), household size (1 or 2, 3 or 4, 5 or 6, and ≥7), employment status (employed, unemployed, homemaker, retired, and unable to work). In the univariate analyses, all of these covariates were significantly associated with homeownership and significantly associated with the number of mentally unhealthy days. We considered these covariates as confounders in the relation between homeownership and number of unhealthy days and therefore included them in our multivariate models.

We used Stata version 12 (StataCorp LP, College Station, Texas) to perform all statistical analyses and take into account the complex sampling design of the survey.

## Results

Among adults aged 35 years or older, about four-fifths (79.3%) owned a home (Table 1). The mean number of mentally unhealthy days was 3.3 days and the median was 0 days, indicating a positive skew. An exact Poisson distribution having a mean of 3.3 days predicted that about 4% of the participants would have zero unhealthy days during the 30-day time frame. However, about two-thirds of individuals (66.8%) reported no mentally unhealthy days, indicating an excess of zeros. The variance was 58.7, which is much greater than the mean (3.3 d).

**Table 1:** Characteristics of Adults Aged 35 or Older in 12 States^a^, 2009 Behavioral Risk Factor Surveillance System

Characteristic	Unweighted Sample Size	%^b^ (95% CI)^c^
**Age group, y**		
35–44	9,034	26.8 (26.0–27.7)
45–54	13,997	27.7 (26.9–28.6)
55–64	15,281	21.8 (21.1–22.5)
≥65	21,251	23.7 (23.0–24.3)
**Sex**		
Male	22,568	47.7 (46.8–48.6)
Female	36,995	52.3 (51.4–53.2)
**Race/ethnicity**		
Non-Hispanic white	43,901	66.8 (65.8–67.8)
Non-Hispanic black	6,008	8.8 (8.3–9.3)
Hispanic	3,399	15.4 (14.5–16.3)
Other	6,255	9.0 (8.4–9.6)
**Education level**		
<High school	5,575	11.6 (10.9–12.4)
High school graduate to <4 y of college	34,130	51.0 (50.1–51.9)
≥4 y of college	19,858	37.4 (36.5–38.2)
**Household income, $**		
<25,000	15,262	22.6 (21.8–23.4)
25,000 to <50,000	15,006	22.7 (21.9–23.4)
≥50,000	22,713	47.2 (46.3–48.1)
Unknown	6,582	7.5 (7.2–7.9)
**Marital status**		
Married	34,624	68.9 (68.1–69.7)
Divorced, widowed, or separated	19,373	21.1 (20.5–21.8)
Never married	5,566	10. 0 (9.4–10.6)
**No. of people in household**		
1 or 2	18,104	14.4 (14.0–14.8)
3 or 4	31,618	52.5 (51.6–53.4)
5 or 6	8,346	26.4 (25.6–27.3)
7 or more	1,495	6.7 (6.0–7.4)
**Employment status**		
Employed	29,110	56.3 (55.4–57.1)
Unemployed	2,879	7.0 (6.5–7.6)
Homemaker	4,259	8.2 (7.7–8.7)
Retired	18,785	21.6 (21.0–22.3)
Unable to work	4,530	6.9 (6.5–7.4)
**Homeownership**		
Own	49,574	79.3 (78.5–80.2)
Do not own	9,989	20.7 (19.8–21.5)
**No. of mentally unhealthy days**		
0	42,029	66.8 (65.9–67.6)
1–10	11,285	22.2 (21.5–23.0)
11–20	2,587	5.0 (4.6–5.4)
21–30	3,662	6.0 (5.6–6.5)

Abbreviations: YEAH, Youth Engagement and Action for Health; SD, standard deviation.

a Alabama, Arkansas, California, Hawaii, Illinois, Kansas, Louisiana, Nebraska, New Mexico, Oklahoma, South Carolina, and Wisconsin.

b Weighted percentage.

c Weighted 95% confidence interval.

The logistic regression analysis found no significant association (*P* = 0.22) between homeownership and having 14 or more mentally unhealthy days in the previous month (Table 2). The parameter estimate (regression coefficient) of homeownership was −0.139 (adjusted odds ratio = 0.87, 95% confidence interval [CI], 0.70–1.09).

**Table 2:** Comparison of Regression Models^a^ in Examining the Association Between Homeownership and Number of Mentally Unhealthy Days in the Previous Month, 2009 Behavioral Risk Factor Surveillance System From 12 States^b^

Regression Model	Parameter Estimate	Standard Error	*P* Value
Model 1: Logistic (≥14 d vs <14 d)	−0.139	(0.113)	.22
Model 2: Linear	−0.456	(0.257)	.08
Model 3: Poisson	−0.085	(0.059)	.15
Model 4: Negative binomial	−0.137	(0.065)	.04
Model 5: Zero-inflated negative binomial			
Zero-inflated component	0.216	(0.072)	.003
Negative binomial component	−0.011	(0.050)	.83

a Non-homeowner is the reference group in all models. All models included the following covariates: age groups, sex, race/ethnicity, education, household income, marital status, household size, and employment status.

b Alabama, Arkansas, California, Hawaii, Illinois, Kansas, Louisiana, Nebraska, New Mexico, Oklahoma, South Carolina, and Wisconsin.

**Figure 1:**
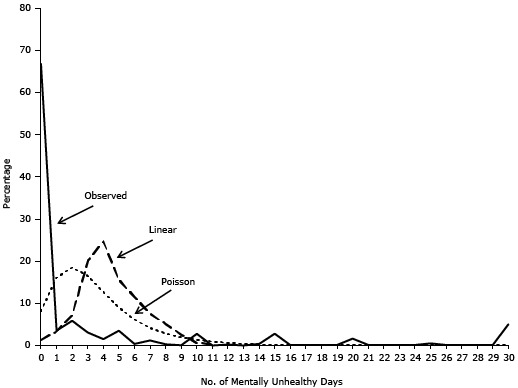
Comparison of the observed percentage distribution of number of mentally unhealthy days and the percentage distribution predicted by the multivariate linear and Poisson regression models. Data were obtained from the 2009 Behavioral Risk Factor Surveillance System in 12 states. **Table:** 

Both linear and Poisson regression models underestimated the percentage of nonoccurrence (0 days) and overestimated the percentage in the category 1 to 9 days (Figure 1). The parameter estimates (regression coefficients) of homeownership in these 2 models were not significantly different from zero (Table 2), indicating homeownership was not significantly associated with the number of mentally unhealthy days in either model.

No. of Mentally Unhealthy Days	Observed	Linear	Poisson
0	66.78	1.37	8.06
1	3.54	3.37	16.04
2	5.89	7.25	18.53
3	3.07	20.09	16.48
4	1.51	24.74	12.66
5	3.51	15.55	8.99
6	0.38	11.53	6.16
7	1.21	7.49	4.19
8	0.28	5.17	2.86
9	0.06	2.59	1.97
10	2.78	0.67	1.36
11	0.01	0.20	0.93
12	0.23	—	0.63
13	0.01	—	0.42
14	0.39	—	0.27
15	2.75	—	0.17
16	0.01	—	0.10
17	0.00	—	0.06
18	0.01	—	0.04
19	0.00	—	0.02
20	1.57	—	0.01
21	0.07	—	0.01
22	0.02	—	0.00
23	0.02	—	0.00
24	0.01	—	0.00
25	0.45	—	0.00
26	0.09	—	0.00
27	0.09	—	0.00
28	0.11	—	0.00
29	0.11	—	0.00
30	5.02	—	0.00

**Figure 2:**
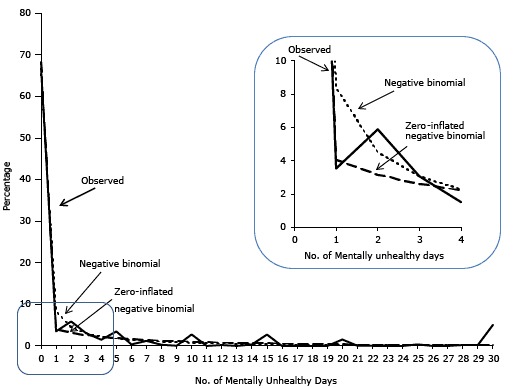
Comparison of the observed percentage distribution of number of mentally unhealthy days and the percentage distribution predicted by the negative binomial and zero-inflated negative binomial models. Data were obtained from the 2009 Behavioral Risk Factor Surveillance System in 12 states. **Table:** 

Negative binomial regression resulted in a better fit of the data than did either linear or Poisson regression (Figure 2). The overdispersion parameter (α) in the negative binomial model was 7.2, which is significantly greater than zero (*P* < .001), indicating that the data were overdispersed. The likelihood-ratio test was 430,000 (*P* < .001), suggesting that negative binomial regression is preferred over Poisson regression. The parameter estimate of homeownership was −0.137 in the negative binomial model (Table 2) (ie, an adjusted rate ratio of 0.87 [exponential (−0.137)] [95% CI, 0.77–0.99]). Hence, homeowners had about 13% fewer mentally unhealthy days than nonowners (*P* = .04). 

No. of Mentally Unhealthy Days	Observed	Negative Binomial	Zero-Inflated Negative Binomial
0	66.78	65.52	68.22
1	3.54	8.38	4.06
2	5.89	4.52	3.16
3	3.07	3.06	2.61
4	1.51	2.28	2.22
5	3.51	1.80	1.92
6	0.38	1.47	1.67
7	1.21	1.23	1.48
8	0.28	1.04	1.31
9	0.06	0.90	1.17
10	2.78	0.79	1.04
11	0.01	0.69	0.94
12	0.23	0.62	0.84
13	0.01	0.55	0.76
14	0.39	0.49	0.69
15	2.75	0.45	0.63
16	0.01	0.40	0.57
17	0.00	0.37	0.52
18	0.01	0.34	0.47
19	0.00	0.31	0.43
20	1.57	0.28	0.39
21	0.07	0.26	0.36
22	0.02	0.24	0.33
23	0.02	0.22	0.30
24	0.01	0.21	0.28
25	0.45	0.19	0.26
26	0.09	0.18	0.24
27	0.09	0.17	0.22
28	0.11	0.15	0.20
29	0.11	0.14	0.19
30	5.02	0.13	0.17

The zero-inflated negative binomial regression provided a better fit of the data than did negative binomial regression (Figure 2). The *z* value of the Vuong test was 42.5 (*P* < .001), confirming that the zero-inflated model fit the data better than the non-zero–inflated model. The parameter estimate in the logistic component of the model was 0.216 (*P* = .003) (Table 2); as such, we can interpret the estimate as an adjusted odds ratio of 1.24 [exponential (0.216)] (95% CI, 1.08–1.43). Hence, homeowners were 24% more likely than non-homeowners to have excess zero mentally unhealthy days. The parameter estimate in the negative binomial component of the model was −0.011 (*P* = 0.83) (ie, an adjusted rate ratio of 0.99 [exponential (−0.011)] [95% CI, 0.90–1.09]), suggesting no significant association between homeownership and the number of unhealthy days. 

## Discussion

In studying the association between homeownership and CDC’s Healthy Days measure as an example, we demonstrated how different models can influence statistical inference — the process of drawing conclusions from empirical data. We did not find an independent association between homeownership and number of mentally unhealthy days by logistic, linear, or Poisson regression models. The negative binomial model showed that homeowners had a moderate but significantly lower number of unhealthy days than non-homeowners. The zero-inflated negative binomial model indicated an association between homeownership and whether individuals reported any mentally unhealthy days but not the number of unhealthy days.

We found that a zero-inflated negative binomial model fit the observed number of mentally unhealthy days reported in BRFSS data better than any of the other models we tested. Despite its ability to model count data, Poisson regression did not fully address the problem of overdispersion. Overdispersion may result in misleading inferences about regression parameters (18). Likewise, negative binomial regression may be less able than zero-inflated negative binomial regression to address the problem of excess zeros. We did not test all possible models in this study. Other models (eg, Hurdle regression, zero-inflated Poisson) can be used to model count data, and there are many methodological deviations of the models we applied (18). Researchers should ensure that their analytic methods fit the data and also use statistical techniques that lead to meaningful interpretations (25). For example, a researcher may find that a zero-inflated negative binomial distribution best fits the data but that a negative binomial distribution without the zero-inflation also meets all statistical assumptions and lends itself to more practical interpretations. In such cases, we advise that researchers consider parsimony and practical interpretation of a model when choosing an analytical method.

The main purpose of this data analysis was not to establish or affirm the “true” relationships between homeownership and number of mentally unhealthy days. We applied various models to BRFSS Healthy Days data as an example to illustrate the importance of appropriate model selection. The study has several limitations. First, it was based on self-reported data from 12 states that elected to include the social context module in its 2009 BRFSS. Second, the survey was conducted through telephone interviews; people without telephones and those who used only cell phones were excluded; these people may be less likely to be homeowners. Third, the BRFSS is a cross-sectional survey: information on the outcome measure (number of mentally unhealthy days) and characteristics (eg, homeownership) of the respondents were assessed at a single point in time. Hence, determining whether the association of characteristics with outcomes preceded or followed the outcomes was not possible.

Any statistical inference requires some assumptions, and incorrect assumptions can invalidate statistical inference (26). Some researchers may ignore the underlying assumptions of their statistical approaches or select a simpler or familiar method as long as the results support their hypothesis. These approaches go against the primary goal of observational epidemiology, which is to assess the detail, strength, direction, shape, and pattern of the relationships between exposures and outcomes. This goal cannot be accomplished without using appropriate statistical methods.

We believe that when the assumptions of analytic techniques are carefully matched to the nature of the data distribution, the results will be more accurate and compelling. False results can mislead researchers, the public, and policy makers and are potentially detrimental to public health. The selection of data analytic techniques is not a trivial statistical matter. Using appropriate analytic procedures will maximize the accuracy and utility of the findings on factors that are of great importance in clinical, policy, and fiscal decisions.

## Figures and Tables

**Table:** 

**1. The activity supported the learning objectives.**				
**Strongly Disagree**				**Strongly Agree**
1	2	3	4	5
**2. The material was organized clearly for learning to occur.**				
**Strongly Disagree**				**Strongly Agree**
1	2	3	4	5
**3. The content learned from this activity will impact my practice.**				
**Strongly Disagree**				**Strongly Agree**
1	2	3	4	5
**4. The activity was presented objectively and free of commercial bias.**				
**Strongly Disagree**				**Strongly Agree**
1	2	3	4	5

## Post-Test Information

To obtain credit, you should first read the journal article. After reading the article, you should be able to answer the following, related, multiple-choice questions. To complete the questions (with a minimum 70% passing score) and earn continuing medical education (CME) credit, please go to http://www.medscape.org/journal/pcd. Credit cannot be obtained for tests completed on paper, although you may use the worksheet below to keep a record of your answers. You must be a registered user on Medscape.org. If you are not registered on Medscape.org, please click on the "Register" link on the right hand side of the website to register. Only one answer is correct for each question. Once you successfully answer all post-test questions you will be able to view and/or print your certificate. For questions regarding the content of this activity, contact the accredited provider, CME@medscape.net. For technical assistance, contact CME@webmd.net. American Medical Association's Physician's Recognition Award (AMA PRA) credits are accepted in the US as evidence of participation in CME activities. For further information on this award, please refer to http://www.ama-assn.org/ama/pub/about-ama/awards/ama-physicians-recognition-award.page. The AMA has determined that physicians not licensed in the US who participate in this CME activity are eligible for **AMA PRA Category 1 Credits™**. Through agreements that the AMA has made with agencies in some countries, AMA PRA credit may be acceptable as evidence of participation in CME activities. If you are not licensed in the US, please complete the questions online, print the AMA PRA CME credit certificate and present it to your national medical association for review.

## Post-Test Questions

### Article Title: Models for Count Data With an Application to Healthy Days Measures: Are You Driving in Screws With a Hammer?

**CME Questions**

1. Which of the following statements regarding different models of data analysis is ***most*** accurate?
  - Logistic regression evaluates data on a continuum of the complete scale of values
  - Ordinary least-squares linear regression is the primary model for count data
  - Poisson regression is the simplest model for count data
  - Zero-inflated negative binomial regression cannot allow for count data that are skewed
2. What is the most common answer from patients regarding the number of poor health days per month on the Behavioral Risk Factor Surveillance System (BRFSS) survey?
  - 0
  - 6
  - 10
  - 14
3. Which of the following statements regarding the results of different data analysis tools is ***most*** accurate?
  - The Poisson regression analysis correctly predicted that 3% of participants had no mentally unhealthy days
  - Linear and Poisson regression models overestimated the percentage with no mentally unhealthy days and underestimated the proportion of participants with 1 to 9 unhealthy days
  - Home ownership failed to affect the percentage of mentally unhealthy disease days in all study analyses
  - The zero-inflated negative binomial regression model provided a better fit of the data compared with negative binomial regression

**Evaluation**
